# Trait Reappraisal Predicts Affective Reactivity to Daily Positive and Negative Events

**DOI:** 10.3389/fpsyg.2016.01000

**Published:** 2016-06-28

**Authors:** Gul Gunaydin, Emre Selcuk, Anthony D. Ong

**Affiliations:** ^1^Department of Psychology, Bilkent UniversityAnkara, Turkey; ^2^Department of Psychology, Middle East Technical UniversityAnkara, Turkey; ^3^Department of Human Development, Cornell University, IthacaNY, USA

**Keywords:** reappraisal, affective reactivity, daily diary designs, emotion regulation, daily life, neuroticism

## Abstract

Past research on emotion regulation has provided evidence that cognitive reappraisal predicts reactivity to affective stimuli and challenge tests in laboratory settings. However, little is known about how trait reappraisal might contribute to affective reactivity to everyday positive and negative events. Using a large, life-span sample of adults (*N* = 1755), the present study addressed this important gap in the literature. Respondents completed a measure of trait reappraisal and reported on their daily experiences of positive and negative events and positive and negative affect for eight consecutive days. Results showed that trait reappraisal predicted lower increases in negative affect in response to daily negative events and lower increases in positive affect in response to daily positive events. These findings advance our understanding of the role of reappraisal in emotion regulation by showing how individual differences in the use of this strategy relate to emotional reactions to both positive and negative events outside the laboratory.

## Introduction

Extant research on emotion regulation has demonstrated that cognitive reappraisal robustly predicts dampened reactivity to affective stimuli and stressful experiences in laboratory settings (Gross and John, 2003; John and Gross, 2004). Experimentally manipulating reappraisal by instructing participants “to adopt a detached and unemotional attitude” toward positive and negative photographs has been shown to be an effective regulation strategy for both positive and negative emotion (e.g., Gruber et al., 2014). Moreover, individual differences in reappraisal ability (i.e., trait reappraisal) are associated with less negative affect following stressful laboratory tasks such as anger provocation (e.g., Memedovic et al., 2010). To date, most research on reappraisal has been conducted in laboratory settings. Whether individual differences in reappraisal predicts affective reactivity to daily life events—especially pleasant experiences—has not been much studied. Although a large body of work has investigated how individuals appraise stressors (e.g., Lazarus and Folkman, 1987) and use various strategies to cope with them including mindfulness exercises (e.g., Grossman et al., 2004), expressive writing (e.g., Pennebaker, 1997), and reappraisal tactics (e.g., Ong et al., 2010), most of this work has not focused on daily affective reactivity. Establishing the link between trait reappraisal and daily affective reactivity is important because (a) the dynamics of affect in day-to-day life are different from those that arise in laboratory tasks and (b) recent prospective studies have shown that daily affective reactivity predicts important psychological and health outcomes—such as psychological well-being (Selcuk et al., 2016), sleep efficiency (Ong et al., 2013), inflammation (Sin et al., 2015), affective disorders (Peeters et al., 2003; Charles et al., 2013), and even mortality (Mroczek et al., 2015). Thus, the aim of the present study was to investigate affective reactivity more dynamically to complement single assessment surveys and laboratory research. To this end, we examined whether trait reappraisal predicts daily affective reactivity to both positive and negative events.

### Reappraisal and Daily Affective Reactivity

Based on Gross’s process model of emotion regulation (Gross, 2001), cognitive reappraisal is a strategy aimed at changing emotional reactions by re-interpreting emotional events or changing the way one thinks about events. Individuals might achieve this by re-evaluating emotional events in an objective, detached, and unemotional manner (e.g., Gross, 1998) or in a more positive light (e.g., Wrosch et al., 2000). Individual differences in reappraisal ability (i.e., trait reappraisal) were shown to modestly predict spontaneous use of reappraisal to cope with stressors. Specifically, individuals high in trait reappraisal also reported spontaneously using this strategy to a greater extent in stressful situations—such as giving a public speech or visiting the dentist (Egloff et al., 2006).

Given reappraisal involves changing the way one thinks about events, a natural implication of this strategy is that individuals might use reappraisal to up- or down-regulate both positive and negative affective reactions. Consistent with this idea, laboratory inductions of reappraisal have either instructed participants to up-regulate (Kim and Hamann, 2007; Ray et al., 2010) or down-regulate (Winecoff et al., 2011; Gruber et al., 2014) affective reactions to positive or negative stimuli (e.g., film clips, pictures). This research demonstrates that participants are able to increase or decrease emotional responses to both positive and negative stimuli when instructed to do so. However, this work does not address the question of whether individuals high in trait reappraisal spontaneously tend to up-regulate or down-regulate emotional reactions to positive and negative experiences.

A few studies have examined the link between trait reappraisal and affective reactivity to laboratory stressors (Mauss et al., 2007; Memedovic et al., 2010; see also Ong et al., 2006). For example, in one study, individuals high (vs. low) in trait reappraisal reported experiencing less anger after receiving insulting feedback in the laboratory (Memedovic et al., 2010). Similarly, spontaneous use of a reappraisal strategy was also shown to be associated with reduced negative affect during a public speaking task (Egloff et al., 2006). Only a few recent studies have investigated the role of reappraisal in coping with day-to-day stressors. In an experience sampling study, Heiy and Cheavens (2014) asked individuals to report at random times throughout the day which strategies they used to regulate affective reactions and found that individuals who reported using reappraisal to regulate negative affect reported better current mood. However, this study focused on participants’ introspections about emotion regulation strategies rather than the extent to which participants used reappraisal to regulate emotional reactions on a regular basis. Other research using similar methodology that controlled for the use of other emotion regulation strategies (e.g., reflection, distraction) failed to find a significant relationship between daily use of reappraisal and negative affect (Brans et al., 2013). However, this research focused on the predictive role of emotion regulation strategies in negative affect rather than in negative affective reactivity to stressors. Another study experimentally manipulated the daily use of reappraisal in response to the most negative event of the day over the course of a week (Ng and Diener, 2013). Results showed that participants who were instructed to reappraise the daily negative event reported lower negative affect about the event compared with participants who were instructed to focus on the event or those who did not try to regulate their emotions. However, this research focused on the instructed use of a reappraisal strategy rather than individual differences in reappraisal. Thus, although research in this area is growing, the paucity of current evidence necessitates continued investigations into the role of trait reappraisal in affective reactivity to daily negative events.

Research evidence linking trait reappraisal to reactions to positive events is even much more scant. Although one study found that individuals who reported using a reappraisal strategy to regulate positive affect did not experience significant changes in mood (Heiy and Cheavens, 2014), this study assessed spontaneous use of reappraisal rather than trait reappraisal. Moreover, unlike regulation of negative affect, it is unclear whether emotional reactions would be spontaneously up- or down-regulated in response to pleasant experiences. On the one hand, high reappraisers might re-interpret a daily positive event as even more pleasant, in which case they would experience greater positive affect (and less negative affect), which is considered an indicator of greater hedonic well-being (Lucas et al., 1996). On the other hand, there is evidence showing that greater positive affective reactivity (Peeters et al., 2003) and lower negative affective reactivity (Bylsma et al., 2011) to positive events are associated with greater depression. Moreover, recent research demonstrates that thinking about an emotional event from the perspective of an outside observer (vs. through one’s own eyes), which is an emotion regulation strategy conducive to reappraisal, reduces the duration of affective experiences while reflecting on both negative and positive events (Verduyn et al., 2012). This work suggests that trait reappraisal might reduce reactivity not only to stressors but also to pleasant events. Therefore, further research is needed to understand how individuals high in trait reappraisal would respond to positive daily experiences.

### Present Research

The aim of the present study was to address these important gaps in the literature by examining whether trait reappraisal predicts daily affective reactivity to both positive and negative events. Toward this aim, we used a large, life-span sample of adults (*N* = 1755) who completed a measure of trait reappraisal and reported on their daily experiences of positive and negative events as well as positive and negative affect for eight consecutive days. This allowed us to investigate whether trait reappraisal predicts changes in positive and negative affect from a day on which participants did not experience a negative (or a positive) event to a day on which they did experience such events (i.e., affective reactivity). We also aimed to investigate the unique role of trait reappraisal in affective reactivity by controlling for individual difference factors that prior work has shown to be linked with affective experience (e.g., Bolger and Zuckerman, 1995; Gunthert et al., 1999; Mroczek and Almeida, 2004; Leger et al., 2016). Toward this aim, we first tested in separate analyses whether age, gender, and neuroticism predicted daily positive and negative affective reactivity to positive and negative events. We then built two final analytical models, one for daily negative affect and one for daily positive affect. The final model for daily negative affect included all variables significantly associated with negative affective reactivity. Likewise, the final model for daily positive affect included all variables that were significantly associated with positive affective reactivity (see Leger et al., 2016 for a similar approach in modeling daily affective reactivity). This approach allowed us to control for potential confounds that may account for the associations between trait reappraisal and affective reactivity.

## Materials and Methods

### Sample and Procedures

Data for the present study came from the second wave of the Midlife Development in the United States (MIDUS; Ryff et al., 2007) and the National Study of Daily Experiences (NSDE; Ryff and Almeida, 2010) projects. The MIDUS project was launched in 1994 (*N* = 7,108) to investigate age-related changes in physical and mental health of adults in the United States. MIDUS II (*N* = 4,963) was conducted in 2004–2006 as a 10-year follow-up on MIDUS I measures and included a phone interview followed by a self-administered questionnaire. Upon completion of MIDUS II, a subset of the MIDUS project participants were recruited to the NSDE II (*n* = 2,022) where they completed an 8-day diary study on common daily positive and negative events, and daily affect. A total of 1,772 adults completed both the MIDUS II and NSDE II studies. Of these, 17 (0.96%) did not complete measures of reappraisal and/or neuroticism, leaving a final analytic sample of 1,755 adults (57% females; mean age = 57 years, range = 33–84 years). Mean time lag between the MIDUS II phone interview and the NSDE II was 21 months (*SD* = 14 months) in the current sample. The present sample was not significantly different than the remainder of the MIDUS II sample in terms of gender composition (χ^2^ = 2.251, *p* = 0.134) or mean age (*t* = 1.687, *p* = 0.092). However, participants in the current sample scored slightly higher in reappraisal (*M* = 3.096, *SE* = 0.014 vs. *M* = 3.048, *SE* = 0.013, *t* = 2.464, *p* = 0.014, *d* = 0.079) and slightly lower in neuroticism (*M* = 2.057, *SE* = 0.015 vs. *M* = 2.122, *SE* = 0.013, *t* = 3.245, *p* = 0.001, *d* = 0.103).

Data collection was reviewed and approved by the Education and Social/Behavioral Sciences and the Health Sciences Institutional Review Boards (IRBs) at the University of Wisconsin–Madison. All participants provided verbal consent. The consent procedure assured participants that their participation was voluntary and that their data would be kept confidential. The IRBs approved the waiver of written consent. Data and documentation for MIDUS II are publicly available at the Inter-university Consortium for Political and Social Research website (ICPSR^1^).

### Measures

#### Trait Reappraisal

Trait reappraisal was measured using the 4-item Positive Reappraisal Scale (Wrosch et al., 2000). Participants indicated the extent to which they used reappraisal strategies to cope with difficult situations (e.g., “When I am faced with a bad situation, it helps to find a different way of looking at things”). Participants responded to the items on a 4-point Likert scale (1 = *A lot* to 4 = *Not at all*). Items were reverse scored so that greater scores reflected higher trait reappraisal. A trait reappraisal score was calculated by averaging across the items (*M* = 3.096, *SD* = 0.604, α = 0.78).

#### Daily Positive and Negative Events

On each of the 8 days during the NSDE II, participants completed a measure of common daily positive and negative events. *Occurrence of daily negative events* was measured using the Daily Inventory of Stressful Events (Almeida et al., 2002). This measure asks participants to indicate whether they had experienced any of the following common daily stressors: an interpersonal conflict, a situation that could end in an argument but they decided to avoid, a problem at work, a problem at home, something bad happening to a close other, perceived discrimination, and any other stressful experiences not covered by the previous categories. Participants also completed a measure of *occurrence of daily positive events* asking whether they had experienced any of the following events each day: a positive interaction with someone, a positive event at work, a positive event at home, something good happening to a close other, and any other pleasant events not covered by the previous categories. Mean number of stressors and positive events experienced per day was 0.53 (*SD* = 0.46) and 1.13 (*SD* = 0.67), respectively.

#### Daily Affect

Participants also indicated the frequency with which they had experienced several affective states each day (0 = *None of the time* to 4 = *All of the time*). The items were adapted from the Non-Specific Psychological Distress and Positive Emotions Scale (Kessler et al., 2002). The items of the negative affect subscale included “restless or fidgety,” “nervous,” “worthless,” “so sad nothing cheer you up,” “everything was an effort,” “hopeless,” “lonely,” “afraid,” “jittery,” “irritable,” “ashamed,” “upset,” “angry,” and “frustrated.” (average α across days = 0.84). The items of the positive affect subscale included “in good spirits,” “cheerful,” “extremely happy,” “calm and peaceful,” “satisfied,” “full of life,” “close to others,” “like you belong,” “enthusiastic,” “attentive,” “proud,” “active,” and “confident.” (average *α* across days = 0.94). Because affect was measured at the day level, mean levels of positive and negative affect were estimated in a two-level null model. Mean daily negative affect was 0.191 (*SE* = 0.006) and mean daily positive affect was 2.727 (*SE* = 0.017).

#### Neuroticism

Neuroticism was measured using the Midlife Development Inventory Personality Scales (Lachman and Weaver, 1997), which were specifically developed for the MIDUS project. The scales were constructed using items from existing well-validated personality inventories, e.g., the Big Five Inventory (John, 1990). Participants were asked to indicate how much each item described them (1 = *A lot* to 4 = *Not at all*). The neuroticism subscale consisted of four items (moody, worrying, nervous, and calm). The item “nervous” overlaps with an item in the daily negative affect scale and the item “calm” overlaps with an item in the daily positive affect scale used in present research. To make sure that any relationship between neuroticism and daily affect was not due to item overlap, these two items were not used when calculating neuroticism scores (see Cacioppo et al., 2010 for a similar approach in estimating the association between loneliness and depression). Responses to the items “moody” and “worrying” were recoded and averaged so that higher scores reflected greater neuroticism (*M* = 2.086, *SD* = 0.715, α = 0.61).

#### Demographic Variables

Participants reported their age and gender at the MIDUS II phone interview.

### Data Analytic Strategy

Daily affective reactivity and its association with trait reappraisal was estimated using multilevel modeling (HLM v7 software). Following prior work (Mroczek et al., 2015; Selcuk et al., 2016), the following two-level model was used to estimate daily negative affect:


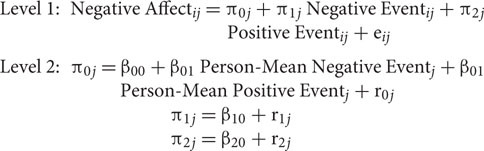


At Level 1, π_0_*_j_* is the intercept and represents negative affect experienced on a day when the participant did not experience a negative or a positive event. Negative Event was a dichotomous variable and was coded either as 0 (when no negative events were experienced) or as 1 (when at least one negative event was experienced). Hence, π_1_*_j_* is the within-person affective reactivity slope corresponding to the difference in the participant’s negative affect on days when at least one negative event was experienced compared to days when no negative events were experienced. In a similar vein, Positive Event was a dichotomous variable indicating whether the participant experienced any positive events that day and its coefficient, π_2_*_j_*, is the within-person affective reactivity slope corresponding to the difference in the participant’s negative affect on days when at least one positive event was experienced compared to days when no positive events were experienced. The error term, e*_ij_*, represented the participant’s deviation from his/her average negative affect. At Level 2, β_00_, β_10_, and β_20_ represent the sample average of negative affect on no-negative event days, negative affective reactivity to stressors, and negative affective reactivity to positive events, respectively. Additionally, β_01_ represents the association between person-mean frequency of negative event exposure and negative affect, and β_02_ represents the association between person-mean frequency of positive events and negative affect. These terms were included in the model to control for between-person differences in negative and positive event exposure. Finally, the error terms, r_0_*_j_*, r_1_*_j_*, and r_2_*_j_* represented deviations from average negative affect, average negative affective reactivity to negative events, and average negative affectivity to positive events, respectively, in the entire sample. Daily positive affective reactivity was estimated in exactly the same way except that the outcome variable was positive affect.

Next, to examine whether reappraisal was associated with affective reactivity, we entered it into the model as a predictor of π_0_*_j_* (estimating the main association of reappraisal with daily affect), π_1_*_j_* (estimating the interaction of negative event exposure and trait reappraisal), and π_2_*_j_* (estimating the interaction of positive event exposure and trait reappraisal). The Level 1 equation of the multilevel model was the same as above and the Level 2 equations were as follows:


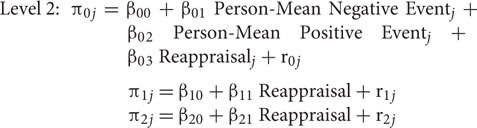


In the above model, β_11_ and β_21_ corresponded to change in affective reactivity to negative events and positive events, respectively, associated with one unit increase in trait reappraisal.

Finally, we examined whether the association between reappraisal and affective reactivity holds after controlling for other individual difference factors associated with daily affect. Following Leger et al. (2016), in separate analyses we first replaced reappraisal in the above Level 2 equations by each of the other individual difference factors (age, gender, and neuroticism) to test whether they predicted affective reactivity slopes. Then, we performed two final models, one for daily positive affect and one for daily negative affect. The final model for daily negative affect included all variables that were significantly associated with negative affective reactivity slopes. Similarly, the final model for daily positive affect included all variables that were significantly associated with positive affective reactivity slopes. In all of the multilevel models, continuous person-level (Level 2) variables were grand-mean centered and robust standard errors were used to estimate the confidence intervals for the model coefficients.

## Results

### Does Trait Reappraisal Predict Daily Negative Affective Reactivity?

**FIGURE 1:**
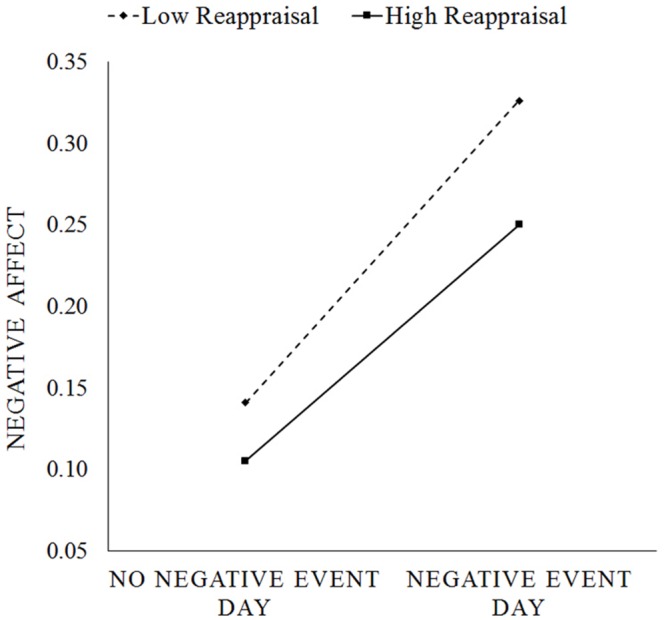
**Change in daily negative affect from a day with no negative events to a day with negative events for high reappraisal (+1 SD of mean) vs. low reappraisal (-1 SD of mean) individuals**.

Multilevel modeling analyses revealed that experiencing a negative event was associated with an increase in negative affect (β_10_ = 0.165, *p* < 0.001, 95% CI = [0.153, 0.177]) whereas experiencing a positive event was not significantly associated with negative affect (β_20_ = 0.002, *p* = 0.647, 95% CI = [-0.008, 0.012]; see Model 1 of **Table 1**). At Level 2, person-mean negative event exposure was positively (β_01_ = 0.158, *p* < 0.001, 95% CI = [0.127, 0.189]) and person-mean positive event exposure was negatively associated with daily negative affect (β_02_ = -0.041, *p* < 0.001, 95% CI = [-0.057, -0.025]), indicating that individuals who on average experience a greater number of stressors and fewer number of pleasant events experience higher daily negative affect. Trait reappraisal was also associated with lower negative affect (β_02_ = -0.026, *p* = 0.010, 95% CI = [-0.046, -0.06]; Model 2 of **Table 1**). It also predicted lower negative affective reactivity to negative event exposure (β_11_ = -0.033, *p* < 0.001, 95% CI = [-0.051, -0.015]). That is, individuals high in reappraisal experienced lower increases in negative affect from a no-stressor day to a day on which they experienced at least one stressful event (**Figure 1**). However, trait reappraisal did not significantly predict negative affective reactivity to positive events (β_21_ = -0.005, *p* = 0.550, 95% CI = [-0.021, 0.011]). Age, gender, and neuroticism were significantly associated with negative affective reactivity to stressful events (all *p*s < 0.005), and age was significantly associated with negative affective reactivity to positive events (*p* = 0.037). Thus, we performed a final model including person-mean negative events, person-mean positive events, reappraisal, neuroticism, age, and gender as predictors of average negative affect (i.e., intercept); trait reappraisal, age, gender, and neuroticism as predictors of negative affective reactivity to stressors; and age as the predictor of negative affective reactivity to positive events. In this final model being female, younger, and higher on neuroticism significantly predicted greater negative affective reactivity to negative events. Trait reappraisal was still significantly associated with negative affective reactivity to negative events in the final model (β_11_ = -0.019, *p* = 0.049, 95% CI = [-0.038, -0.0001]). Age was also negatively associated with negative affective reactivity to positive events (see Model 3 of **Table 1** for all coefficients).

**Table 1:** Multilevel models predicting daily negative affect.

	Model 1		Model 2		Model 3	
Predictors	Coefficient	*p*	Coefficient	*p*	Coefficient	*p*
**Intercept, π_0_**						
Intercept, β_00_	0.123 (0.006)	<0.001	0.123 (0.006)	<0.001	0.121 (0.008)	<0.001
Average negative events, β_01_	0.158 (0.016)	<0.001	0.155 (0.016)	<0.001	0.139 (0.016)	<0.001
Average positive events, β_02_	-0.041 (0.008)	<0.001	-0.034 (0.008)	<0.001	-0.031 (0.008)	<0.001
Reappraisal, β_03_	-	-	-0.026 (0.010)	0.010	-0.014 (0.008)	0.066
Age, β_04_	-	-	-	-	0.001 (0.0004)	0.001
Gender, β_05_	-	-	-	-	0.003 (0.008)	0.716
Neuroticism, β_06_	-	-	-	-	0.054 (0.008)	<0.001
**Negative event slope, π_1_**						
Intercept, β_10_	0.165 (0.006)	<0.001	0.165 (0.006)	<0.001	0.149 (0.007)	<0.001
Reappraisal, β_11_	-	-	-0.033 (0.010)	<0.001	-0.019 (0.010)	0.049
Age, β_12_	-	-	-	-	-0.002 (0.0005)	<0.001
Gender, β_13_	-	-	-	-	0.024 (0.011)	0.022
Neuroticism, β_14_	-	-	-	-	0.050 (0.008)	<0.001
**Positive event slope, π_2_**						
Intercept, β_20_	0.002 (0.005)	0.647	0.002 (0.005)	0.686	0.002 (0.005)	0.732
Reappraisal, β_21_	-	-	-0.005 (0.008)	0.550	-	-
Age, β_22_	-	-	-	-	-0.001 (0.0004)	0.030

*Standard errors are provided in parantheses. All continuous variables were grand-mean centered. For gender, male was coded as 0 and female was coded as 1.*

### Does Trait Reappraisal Predict Daily Positive Affective Reactivity?

**FIGURE 2:**
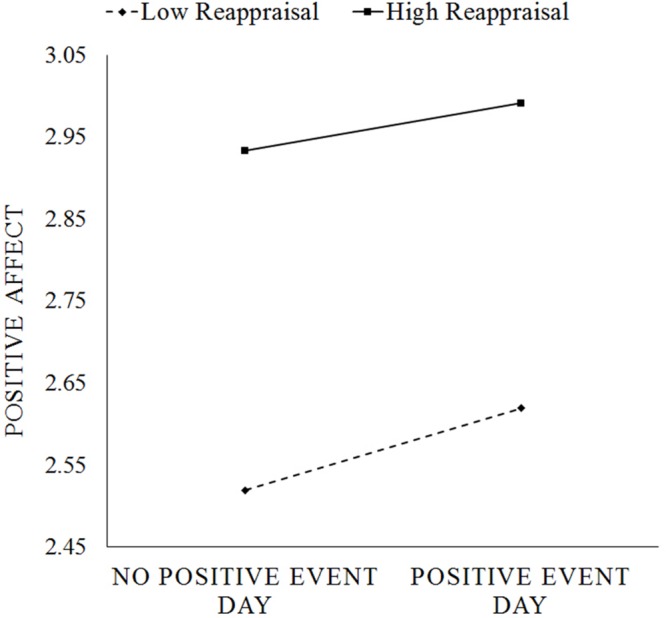
**Change in daily positive affect from a day with no positive events to a day with positive events for high reappraisal (+1 SD of mean) vs. low reappraisal (-1 SD of mean) individuals**.

At the within-person level, exposure to a positive event was associated with increases (β_20_ = 0.081, *p* < 0.001, 95% CI = [0.061, 0.101]) and exposure to a negative event was associated with decreases in daily positive affect (β_10_ = -0.143, *p* < 0.001, 95% CI = [-0.161, -0.125]; Model 1 of **Table 2**). There was also between-person differences in the links between daily events and positive affect. Individuals who experienced on average greater number of positive events and fewer number of negative events experienced higher daily positive affect (β_02_ = 0.240, *p* < 0.001, 95% CI = [0.189, 0.291]; β_01_ = -0.521, *p* < 0.001, 95% CI = [-0.601, -0.441, respectively]. Trait reappraisal also positively predicted daily positive affect (β_03_ = 0.339, *p* < 0.001, 95% CI = [0.278, 0.400]; Model 2 of **Table 2**). Importantly, reappraisal negatively predicted positive affective reactivity to positive events (β_21_ = -0.036, *p* = 0.037, 95% CI = [-0.069, -0.003]). That is, high reappraisal individuals experienced lower increases in positive affect from a no-positive event day to a day in which they experienced at least one positive event (**Figure 2**). However, trait reappraisal did not predict positive affective reactivity to negative events (β_11_ = 0.008, 95% CI = [-0.019, 0.035]. Although gender was significantly associated with positive affective reactivity to both positive events (*p* = 0.050) and negative events (*p* < 0.001), neither age nor neuroticism was significantly associated with positive affective reactivity to positive or negative events (all *p*s > 0.064). Thus, we performed a final model including person-mean negative events, person-mean positive events, reappraisal, neuroticism, age, and gender as predictors of average positive affect (i.e., intercept), gender as the predictor of positive affective reactivity to negative events, and reappraisal and gender as predictors of positive affective reactivity to positive events. In the final model, reappraisal still significantly predicted positive affective reactivity to positive events (β_21_ = -0.037, *p* = 0.031, 95% CI = [-0.070, -0.004]). Gender also significantly predicted positive affective reactivity, with female (vs. male) participants’ positive affective reactivity being higher in response to positive events and lower in response to negative events (see Model 3 of **Table 2** for all coefficients).

**Table 2:** Multilevel models predicting daily positive affect.

	Model 1		Model 2		Model 3	
Predictors	Coefficient	*p*	Coefficient	*p*	Coefficient	*p*
**Intercept, π_0_**						
Intercept, β_00_	2.723 (0.019)	<0.001	2.726 (0.018)	<0.001	2.714 (0.026)	<0.001
Average negative events, β_01_	-0.521 (0.041)	<0.001	-0.492 (0.040)	<0.001	-0.409 (0.040)	<0.001
Average positive events, β_02_	0.240 (0.026)	<0.001	0.169 (0.026)	<0.001	0.142 (0.025)	<0.001
Reappraisal, β_03_	-	-	0.339 (0.031)	<0.001	0.291 (0.030)	<0.001
Age, β_04_	-	-	-	-	0.003 (0.001)	0.006
Gender, β_05_	-	-	-	-	0.021 (0.036)	0.549
Neuroticism, β_06_	-	-	-	-	-0.183 (0.023)	<0.001
**Negative event slope, π_1_**						
Intercept, β_10_	-0.143 (0.009)	<0.001	-0.143 (0.009)	<0.001	-0.111 (0.012)	<0.001
Reappraisal, β_11_	-	-	0.008 (0.014)	0.576	-	-
Gender, β_12_	-	-	-	-	-0.056 (0.017)	0.001
**Positive event slope, π_2_**						
Intercept, β_20_	0.081 (0.010)	<0.001	0.079 (0.010)	<0.001	0.055 (0.013)	<0.001
Reappraisal, β_21_	-	-	-0.036 (0.017)	0.037	-0.037 (0.017)	0.031
Gender, β_22_			-	-	0.041 (0.020)	0.035

*Standard errors are provided in parantheses. All continuous variables were grand-mean centered. For gender, male was coded as 0 and female was coded as 1.*

## Discussion

Using multilevel analyses of daily experience data from a life-span sample of adults, the present research showed that trait reappraisal predicted lower increases in negative affect in response to daily negative events. However, trait reappraisal did not significantly predict change in negative affect in response to positive events. The association between trait reappraisal and negative affective reactivity to stressors held controlling for other significant predictors of negative affective reactivity (i.e., age, gender, and neuroticism). These findings contribute to the literature in affect regulation by showing the link between trait reappraisal and negative affective reactivity to stressors in *daily life*. This is a small but practically important effect given that daily affective reactivity is known to play a central role in psychological and physical well-being—from how fulfilled one’s life is (Selcuk et al., 2016), to how well one sleeps (Ong et al., 2013), and ultimately to how long one lives (Mroczek et al., 2015).

The present research also showed that trait reappraisal predicted lower increases in positive affect in response to daily positive (but not negative) events. Although this is a small association, it significantly contributes to theorizing on reappraisal by clarifying whether individual differences in trait reappraisal would be associated with up- or down-regulation of positive affect in response to pleasant events. Given previous studies investigating affective reactivity to positive stimuli almost exclusively focused on the instructed use of the reappraisal strategy (e.g., Gruber et al., 2014), little is known about how individual differences in the use of this strategy relate to affective reactivity to positive stimuli or pleasant daily experiences. Our findings demonstrate that individual differences in reappraisal predict *down*-regulation of positive affect in response to daily positive events, consistent with recent evidence showing that reappraising a positive affect-eliciting event by adopting an observer’s perspective is associated with shorter durations of positive affect (Verduyn et al., 2012). These findings have important clinical implications given that past research has demonstrated that down-regulation of positive affect in response to daily positive events is associated with better mental health (Peeters et al., 2003).

Another important implication of the present research is for understanding potential mechanisms explaining the well-established link between trait reappraisal and psychological adjustment. Specifically, trait reappraisal has been shown to predict lower depression (Garnefski and Kraaij, 2006; Aldao et al., 2010), higher self-esteem (Nezlek and Kuppens, 2008), greater life satisfaction, and better interpersonal relationships (Gross and John, 2003). However, mechanisms explaining these associations have not received much research attention. Our findings suggest that daily affective reactivity might be one of the potential mechanisms explaining the link between higher reappraisal ability and psychological adjustment.

To understand whether the use of affect regulation strategies—including reappraisal—might alter individual differences in daily affective reactivity one important question is: how stable are these individual differences? On the one hand, there is reason to expect stability. Research showed that affective reactivity is linked with relatively stable characteristics—such as activation in frontal regions of the brain (Davidson, 1992) and recollections of childhood experiences (Mallers et al., 2010). On the other hand, there is evidence that contextual factors—such as how overwhelming and uncontrollable individuals view current life demands (Sliwinski et al., 2009) or how responsive they see close relationship partners (Selcuk et al., 2016)—predict affective reactivity. This work suggests that individuals’ affective reactivity might change if their personal circumstances change. Indeed, research using the MIDUS sample showed modest stability (*r* = 0.37) in negative affective reactivity over 10 years (Sliwinski et al., 2009). In other words, although affective reactivity demonstrates some stability over time, there is also room for change. Therefore, it might be possible to alter one’s affective reactivity by repeatedly using adaptive emotion regulation strategies such as reappraising or re-interpreting events (e.g., Gross, 2001), viewing them from a distanced perspective (e.g., Ayduk and Kross, 2010), activating mental representations of significant others (e.g., Selcuk et al., 2012), exercising mindfulness (e.g., Grossman et al., 2004), or writing about one’s deepest thoughts and feelings about emotional events (e.g., Pennebaker, 1997). These are undoubtedly important questions for future research.

In sum, the present research is the first to document the link between trait reappraisal and affective reactivity to both positive and negative experiences in everyday life. Notably, the large sample spanning from middle to late adulthood increases the confidence in the generalizability of the findings. This work advances our understanding of the role of reappraisal in emotion regulation by showing how individual differences in the use of this strategy relate to emotional reactions to *both* stressors and pleasant events *outside the laboratory* and by offering a potential mechanism that may explain why trait reappraisal is associated with better psychological adjustment.

## Author Contributions

GG, ES, and AO developed the study idea. ES and GG conceived the data analytic strategy. ES conducted the data analysis. GG drafted the manuscript, and ES and AO provided critical revisions. All authors approved the final version of the manuscript for submission.

## Conflict of Interest Statement

The authors declare that the research was conducted in the absence of any commercial or financial relationships that could be construed as a potential conflict of interest.

## Footnotes

1 www.icpsr.umich.edu/icpsrweb
